# Total knee arthroplasty and physical therapy for arthropathy in alkaptonuria: A 4-year follow-up case report

**DOI:** 10.3389/fsurg.2022.913120

**Published:** 2023-01-06

**Authors:** Linfeng Wu, Yu Hu

**Affiliations:** ^1^ Department of Orthopedics, The First People's Hospital of Longquanyi District, Chengdu, China; ^2^ Department of Rehabilitation Medicine, The Third People's Hospital of Chengdu, Affiliated Hospital of Southwest Jiaotong University, Chengdu, China

**Keywords:** alkaptonuria (AKU), arthroplasty, ochronosus, arthropathy, physical therapy

## Abstract

**Introduction:**

Alkaptonuria is a rare autosomal recessive metabolic disorder which leads to accumulation of homogentisic acid in the body.

**Case Presentation:**

We report a rare case of an alkaptonuria-related knee arthritis who underwent left total knee arthroplasty and received postoperative systematic physical therapy in a 57-year-old male patient. The patient has suffered from bilateral knee pain for over 4 years. The patient developed melanin pigmentation on the skin of the whole body, especially on the face and auricle. He self-reported that fresh urine was normal color but after standing overnight, the color deepened to black or soy color. He underwent routine urine examination for many times, but no obvious abnormality was found. The patient has suffered from low back pain for more than 20 years. He had been considered for lumbar disc herniation and ankylosing spondylitis after many in-hospital visits. After symptomatic medication, there was no obvious relief. We followed the patient for 4 years after surgery.

**Result:**

The patient presented with pain relief and enhanced range of motion at the 4-year follow-up. The improvements of daily living and the pain relief suggest that the surgery is appropriate for this rare disease.

**Conclusion:**

It is rare that the knee pain is diagnosed as alkaptonuria. After total knee arthroplasty and physical therapy, the patient had a good outcome. This case provides experience for the diagnosis and treatment of alkaptonuria-related knee arthritis.

## Introduction

Alkaptonuria (AKU) is a rare autosomal recessive metabolic disorder caused by deficiency of homogentisate acid 1, 2-dioxygenase due to mutation of 3q21–q23 position base sequence, which leads to accumulation of homogentisic acid (HGA) in the body (1–3). The incidence of AKU in the population is approximately 1 in 100,000 to 250,000 (4). HGA and its oxidation products can accumulate in connective tissues and musculoskeletal tissues of the body, such as joints, tendons, skin, nose and ear cartilage, especially hyaline cartilage which is frequently affected (5). In the early stage of AKU patients, urine darkening and skin pigmentation are the main manifestations. In the late stage, the irreducible urinary HGA erodes the cartilage and soft tissue of the body and causes secondary lesions (6). The weight-bearing joints become brittle due to the erosion of HGA, thus presenting rapidly progressive degenerative joint disease (5).

We report a case of a 57-year-old man with a family history of consanguineous marriage of his parents who was diagnosed with AKU-related knee arthritis and underwent left total knee arthroplasty (TKA), followed by systematic physical therapy.

## Case description

A 57-year-old male patient with height 170 cm, weight 55 kg and body mass index (BMI) 19 was admitted to department of orthopedic for 4 years with bilateral knee pain, especially on the left side. Four years ago, there was no obvious trigger for bilateral knee pain, especially when going up and down stairs and walking more than 300 meters. Two years ago, the pain in both knees exacerbated, especially in the left knee joint. The patient has gradually developed melanin pigmentation on the skin of the whole body since 2 years ago, especially on the face and auricle. The patient's self-reported that fresh urine was normal color, but after standing overnight, the color deepened to black or soy color since childhood. The patient underwent routine urine examination for many times, but no obvious abnormality was found, so no treatment was given. The patient has suffered from low back pain for more than 20 years. He had been considered for lumbar disc herniation and ankylosing spondylitis after many in-hospital visits. After symptomatic medication, there was no obvious relief.

About 2 years ago, the patient developed a spontaneous rupture of the left Achilles tendon without obvious cause, which was sutured surgically. The left Achilles tendon tissue showed a large amount of unidentified black deposit during the surgery.

**Figure 1:**
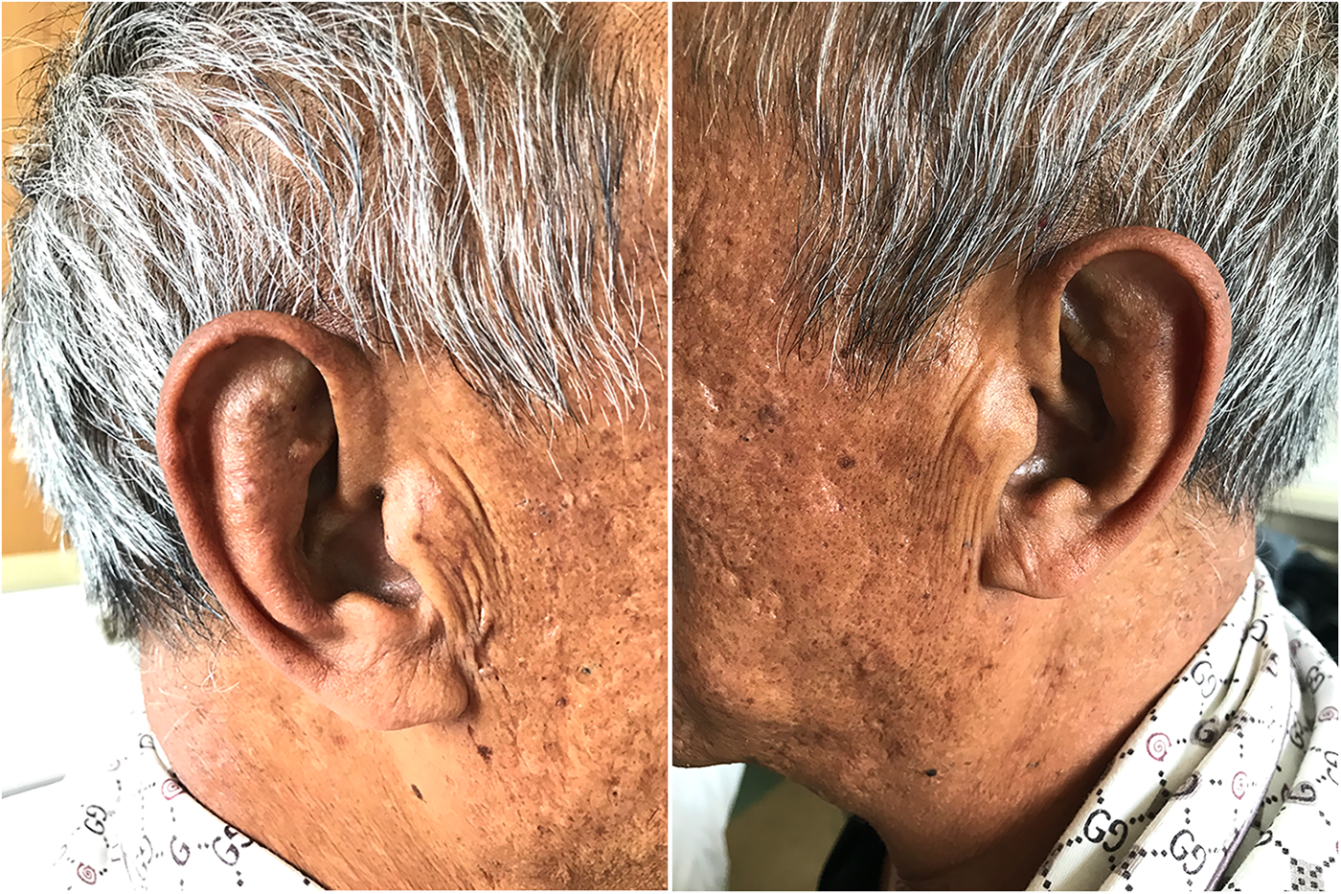
Melanin deposition in the patient's auricle and face.

**Figure 2:**
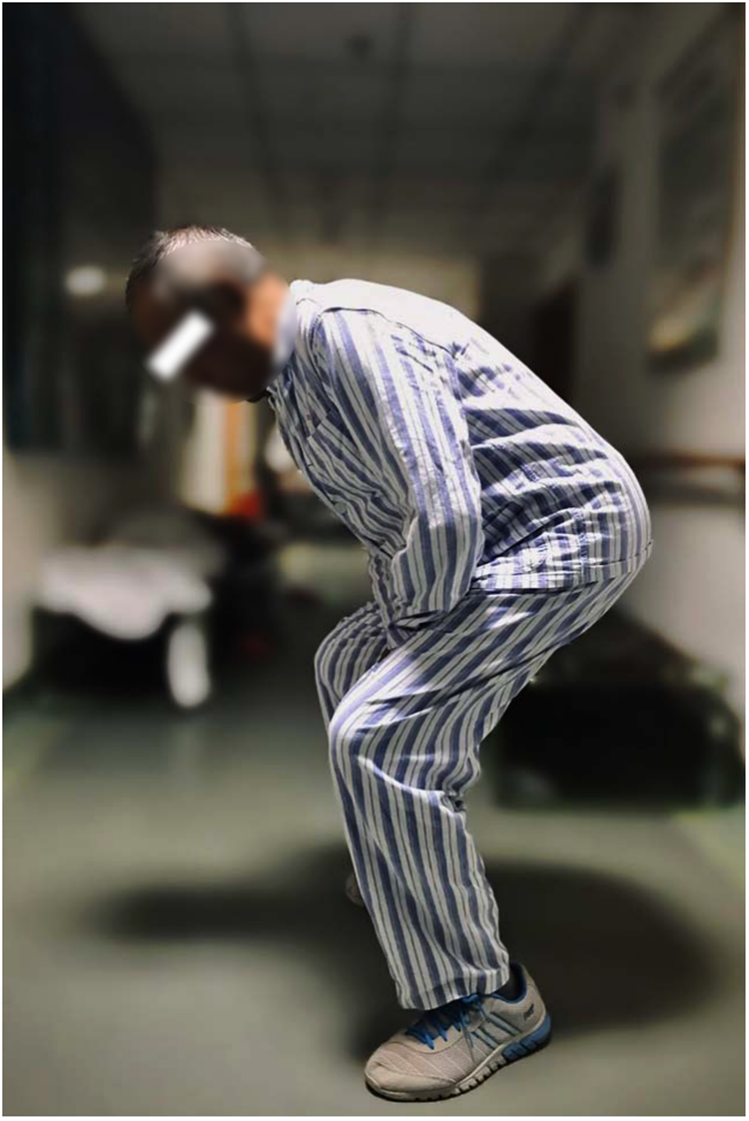
Preoperative hip and knee joint movements.

Physical examination revealed multiple skin melanin deposits, especially on the face and auricle (Figure 1). The flexion, extension and lateral flexion of the spine were significantly limited (Figure 2), the straight leg raising test of both lower extremities were negative. The left knee showed valgus deformity and flexion contracture, with flexion contracture of about 5°, valgus of about 5°, flexion range of motion (ROM) 0–90°, bilateral quadriceps muscle strength grade 4, left knee joint HSS scoring 40 points and 69 points on the left side.

**Figure 3:**
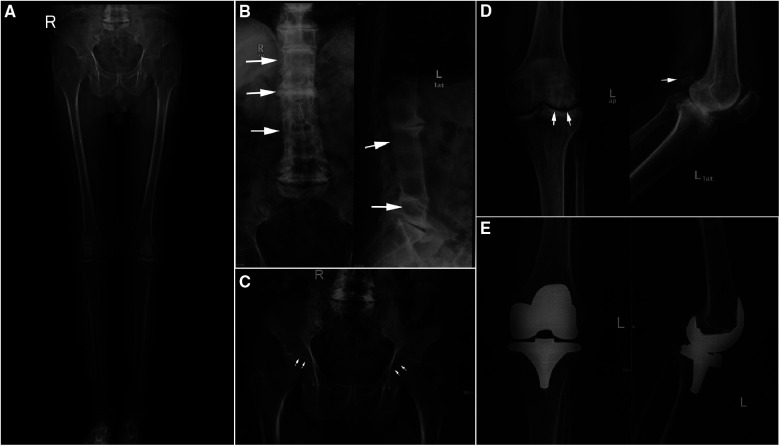
Pre and postoperative digital radiation (DR) image. (**A**) Preoperative full leg weight bearing x-Ray of both lower extremities. (**B**) Preoperative anteroposterior and lateral DR views of lumbar. (**C**) Preoperative anteroposterior DR view of pelvic. (**D**) Preoperative anteroposterior and lateral DR views of left knee. (**E**) Postoperative anteroposterior and lateral DR views of left knee.

Auxiliary examination showed that blood routine, liver and kidney function, erythrocyte sedimentation rate, C-reactive protein, HLA-B27 antigen, interleukin-6, rheumatoid factor, joint fluid examination, and a full set of immune examinations were normal. Radiographically, Lung computerized tomography (CT) showed no obvious abnormality. Digital radiography (DR) showed hyperplasia of bilateral sacroiliac joint surfaces, osteoporosis of both hips and knees, joint space narrowing, intervertebral space narrowing, and vertebral border hyperplasia (Figures 3A–D).

**Figure 4:**
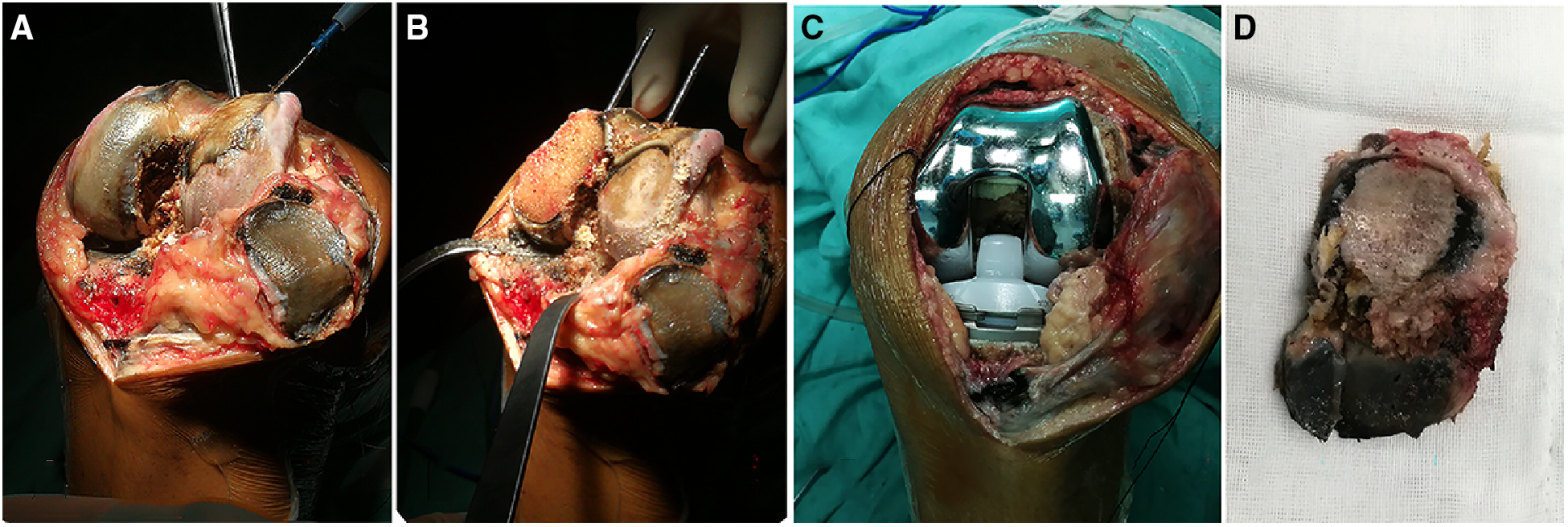
(**A–C**) Melanin deposits on articular cartilage during surgery. (**D**) A bone fragment taken during surgery.

Based on the above examinations, the patient was diagnosed with AKU-related knee arthritis and we considered left-sided TKA. The surgery was performed under general anesthesia. During the surgery, a large amount of black deposit was found in the articular cartilage surface and patellar ligament of the left knee (Figures 4A–D). The surgery lasted for approximately 1 h, and the operative limb was pressures-bandaged postoperatively. Postoperative DR showed that the position of the knee prosthesis was satisfied (Figure 3E).

**Figure 5:**
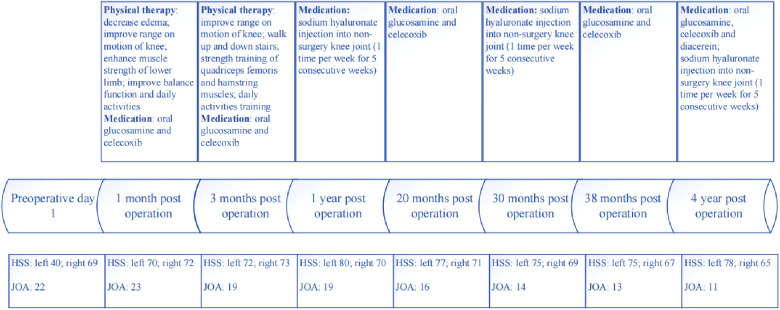
The timeline of full-process of assessment and therapy.

The patient received in-hospital and out-patient physical therapy from the first postoperative day. One week after the surgery, the patient was discharged with a left knee flexion ROM of 95° and a visual analogue scale (VAS) score of 4. Post-operative physical therapy was performed in outpatient for 15 weeks after discharge. Physical therapy focuses on reducing edema, enhancing knee ROM, improving muscle strength of lower extremity, and alleviating gait and balance disorders. Changes in HSS score and VAS score are shown in Table 1. During our 4-year follow-up, the pain in surgery-side knee joint was significantly relieved, but the pain in the contralateral knee joint and low back was gradually increasing year by year. We mainly used HSS and JOA to assess the patient's knee and spine function (Table 2). By the last follow-up day, the patient's activities of daily living were normal, and he could walk without crutches. A single walking distance was about 1 km. For postoperative treatment, we gave patients oral glucosamine and celecoxib for symptomatic treatment outside the hospital, and from the second year after operation, a course of sodium hyaluronate was injected into the contralateral knee joint once a year (1 time per week for 5 consecutive weeks), which temporarily relieved the patient's symptoms, but failed to stop the disease process. The timeline of full-process of assessment and therapy is shown in Figure 5.

**Table 1:** Changes in HSS score and JOA score.

	Preoperation	1 month post operation	3 months post operation	1 year post operation	20 months post operation	30 months post operation	38 months post operation	4 years post operation
HSS	L 40; R 69	L 70; R 72	L 72; R 73	L 80; R 70	L 77; R 71	L 75; R 69	L 75; R 67	L 78; R 65
JOA	22	23	23	19	16	14	13	11

## Discussion

Due to the rarity and low incidence of the disease and the atypical clinical symptoms, it is difficult to make an early and correct diagnosis. In the early stage of the disease, when the degree of damage to joints and soft tissues by HGA and its metabolites is relatively mild, patients may have no obvious symptoms except for skin pigmentation and the changes in urine color (7). However, as AKU progresses and connective tissue is affected, Gil et al. pointed out that the intervertebral discs of the spine may be affected first, followed by the weight-bearing joints of the extremities, which may cause low back pain and degenerative changes in the knee and hip joints (8). However, the symptoms at this stage are not specific, so it is difficult to distinguish it from traditional arthritis and lumbar disc herniation. In the late stage when the systemic fibrous connective tissue is severely eroded, the joints and spine may have obvious narrowing of the joint and intervertebral space and even osseous ankylosis, and therefore there is a high risk of being misdiagnosed as ankylosing spondylitis or rheumatoid arthritis. Therefore, how to diagnose AKU accurately and quickly is crucial. The current gold standard for the diagnosis of AKU is considered to be the detection of HGA in the urine (9).

**Figure 6:**
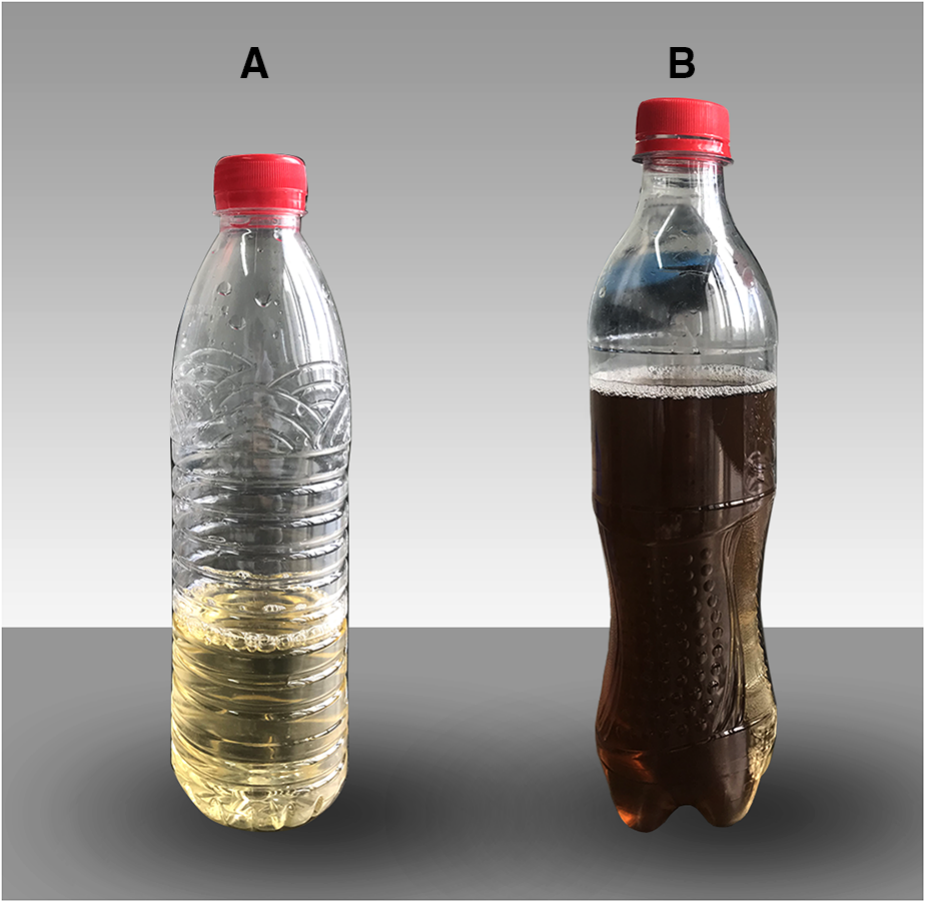
Urine color at different times. (**A**) Fresh urine. (**B**) Urine exposed to air for 24 h.

NaOCl·5H20 has been shown to greatly accelerate the oxidation of HGA, which provides a new idea for the rapid detection and diagnosis of AKU (10). However, this test is not included in routine urine examination. Therefore, most cases can only be recognized and diagnosed by clinical manifestations. In this case, after exposed to air for 24 h, the fresh urine gradually changed from yellow to black (Figure 6). Also, the patient's skin had extensive melanosis, especially on the auricle, sclera, nose, and cheeks. Combined with imaging findings of the destruction of cartilage tissue throughout the body, which caused joint and spinal symptoms, and the melanin deposition of tendon tissue found in the history of Achilles tendon surgery, we comprehensively considered the diagnosis of arthropathy in AKU in this patient. Its symptoms and imaging findings are often difficult to differentiate from rheumatoid arthritis and ankylosing spondylitis, but it should be noted that rheumatoid arthritis is often characterized by symmetrical facet joint pain and morning stiffness, and the positive rheumatoid factor. Additionally, ankylosing spondylitis is often accompanied by the rising HLA-B27, but alkaptonuria does not have these manifestations, and it is believed that this can be used as one of the means of differential diagnosis.

Due to the low incidence of AKU, there are currently no clinical studies with a large sample size on the disease and no unified treatment standard (6). One study has shown that a strict low-protein diet can reduce the production of HGA, thus achieving the purpose of delaying the development of the disease (11). Additionally, a three-year prospective study of 39 patients has confirmed that the use of nitisinone can inhibit the activity of hydroxyphenylalanine oxidase during the formation of HGA, thereby delaying the clinical course of AKU (12). When patients suffer from joint pain, especially knee pain, studies have reported that the use of glucosamine can effectively relieve joint pain (13), but due to the lack of a large number of clinical studies, its clinical effects should be considered with caution. Also, vitamin E and N-acetylcysteine have been shown to be potential treatments because they scavenge free radicals and limit oxidative damage to joint tissue (8). In addition, studies have shown that joint debridement using arthroscopy and sodium hyaluronate injection can also relieve knee pain (14).

Similar to current case, there is a relatively large number of studies showing that joint replacement in patients with ochronotic arthropathy is effective in relieving joint pain and improving activities of daily living (11, 13, 15). Nevertheless, there is also evidence that no matter what the treatment is, it can only delay the progression of the AKU, and cannot achieve the purpose of curing it (16). However, there was a case report that in an AKU patient with advanced cirrhosis with typical clinical and imaging manifestations, HGA was unexpectedly found to disappear in the urine after liver transplantation, suggesting that the disease may be related to liver metabolism (17). Currently, gene therapy has become a focus of attention, and a mouse-related gene defect model has been successfully established, which provides the possibility of a complete cure for the AKU (18).

## Conclusion

In summary, AKU is a rare disease with a very low incidence. The current treatment is based on early diagnosis and symptomatic treatment, and the whole process and comprehensive management should be implemented. Patients who can be diagnosed at an early stage should undergo metabolic intervention to delay the progression of the disease. In AKU patients with advanced ochronotic arthropathy, surgery and postoperative physical therapy appear to be effective in reducing pain and returning to daily activities. This case provides new information critical to studying AKU. As its mechanism remains unclear, further research is needed to reveal it.

## Data Availability

The original contributions presented in the study are included in the article/**Supplementary Material**, further inquiries can be directed to the corresponding author/s.
